# Quantifying the Reduction Intensity of Handaxes with 3D Technology: A Pilot Study on Handaxes in the Danjiangkou Reservoir Region, Central China

**DOI:** 10.1371/journal.pone.0135613

**Published:** 2015-09-02

**Authors:** Hao Li, Kathleen Kuman, Chaorong Li

**Affiliations:** 1 Key Laboratory of Vertebrate Evolution and Human Origins of Chinese Academy of Sciences, Institute of Vertebrate Paleontology and Paleoanthropology, Chinese Academy of Sciences, Beijing, China; 2 School of Geography, Archaeology and Environmental Studies, University of the Witwatersrand, Johannesburg, South Africa; 3 Evolutionary Studies Institute, University of the Witwatersrand, Johannesburg, South Africa; University of Oxford, UNITED KINGDOM

## Abstract

This paper presents an approach to analyzing the reduction intensity of handaxes with the aid of 3D scanning technology. Two quantitative reduction indices, the Scar Density Index (SDI) and the Flaked Area Index (FAI), are applied to handaxes from the third terrace of the Danjiangkou Reservoir Region (DRR), central China, dated to the Middle Pleistocene. The results show that most of the DRR handaxes in this sample show moderate reduction, which also reflects a least-effort reduction strategy and a generally short use-life for these tools. Detailed examination of the DRR handaxes by sector reveals that the tips generally show the most reduction, while the bases show the least shaping, with cortex often preserved on the base to facilitate handling. While western Acheulean assemblages in this regard are variable, there are many examples of handaxes of varying age with trimming of the bases. We also found no significant differences in the levels of reduction between the two main raw materials, quartz phyllite and trachyte. However, the type of blank used (large flakes versus cobbles) and the type of shaping (bifacial, partly bifacial and unifacial) do play a significant role in the reduction intensity of the DRR handaxes. Finally, a small number of handaxes from the younger (the early Late Pleistocene) second terrace of the DRR was compared with those from the third terrace. The results indicate that there is no technological change in the reduction intensity through time in these two DRR terraces.

## Introduction

Since the 1980s, lithic researchers have worked to develop a series of methods to measure reduction intensity, particularly as a tool for interpreting curation or re-sharpening of tools and morphological variability, and they have applied them to both experimental and archaeological assemblages. Among them, the quantitative reduction indices proposed by Dibble [1] (the ratio of the remaining surface area to platform area), and Kuhn [2] (the ratio of flake thickness at the point where retouch scars terminate to maximum medial thickness, also well-known as a Geometric Index of Reduction) are the two most influential indices. These two indices and their modified versions are still frequently applied in current research, which confirms their usefulness [3–20]. Generally speaking, these methods for measuring reduction mainly focus on morphological attributes to estimate reduction intensity. In other words, as reduction continues, the corresponding size, shape and other morphological properties change as well. Shott and Weedman [11] have summarized them as three specific methods, namely, a simple size measurements method, a geometric measurements method, and an allometric method that relates shape and other attributes to size.

Allometry has also influenced the reduction study of handaxes. Based on the reduction hypothesis, McPherron [21] reanalyzed 38 British handaxe assemblages classified by Roe into either pointed or ovate groups. He argued that these shapes actually reflect different reduction intensities, with pointed handaxes in an initial stage of reduction and ovate handaxes in a later stage. For quantitatively measuring the reduction of handaxes, McPherron employed linear measurements, namely the tip length, overall length and width in his study and assumed that pointed handaxes would have both a long tip length and a long overall length. As reduction continued, tip length and length decreased quickly, but width decreased at a lower rate, finally leading to the formation of ovate shaped handaxes with lower elongation values (Length/Width) [21–23]. Thus, through comparing the tip length, length and elongation ratio, McPherron argued that it is possible to estimate the reduction intensity of different handaxe assemblages. This allometric method provided a new perspective in interpretation of the morphological variability of handaxes, and more importantly, it shifted attention from the final form of handaxes to their reduction process and flaking strategies. Consistent with this allometric method, most current reduction intensity analyses of handaxes are now integrated into studies of morphological variability [24–32].

In contrast to McPherron’s analysis of the size and shape of handaxes in relation to re-sharpening, McNabb et al. [33] proposed a method for the technological study of ‘shaping’, without reference to re-sharpening. By recording the extent of secondary flaking (flake scars > 1.5cm in length) and the degree of edge trimming (flake scars < 1.5cm in length), the authors identified five subcategories of secondary flaking for each face of a handaxe and five ordinal scales of edge trimming for each section of a handaxe (see McNabb et al. [33], Figs 4 and 7). The frequencies of these attributes are then classed by the extent of reduction as light, moderate or extensive. This is a detailed and useful approach to reduction analysis of handaxes involving technological attributes. However, a potential problem may lie in the subjective divisions of different types of secondary flaking and different scales of edge trimming, which can vary according to the observer.

Another easily applied approach used by some researchers is the direct counting of the number of scars on a handaxe. Scar numbers are relatively easier to quantify at different stages of reduction for Early Stone Age (Lower Palaeolithic) handaxes than for the generally small-sized tools in the Middle Stone Age (Middle Palaeolithic) and Later Stone Age (Upper Palaeolithic). The assumption here is that extensively retouched handaxes would have more flake scars than less reduced handaxes. Using this concept, Hou et al. [34] compared the number of flake scars on the Bose Large Cutting Tools (LCTs; ~0.803Ma) with the number of scars on LCTs in two western Acheulean assemblages of similar age (0.99Ma-0.7Ma; Olorgesailie Members 1 through 7 in Kenya and Bed IV of Olduvai in Tanzania). They concluded that the Bose LCTs have a similar number of scars as the western Acheulean, and therefore they support the proposal that there is no technological difference between handaxes in the East and West. In addition, in Sharon’s [35] comparative study of handaxes from Africa, West Asia and India, the number of scars was also regarded as an important attribute for the analysis of reduction extent.

Although the counting of flake scars is a useful and easily applied approach, it also has one limitation. As mentioned by Sharon [35], the visible number of flake scars on discarded handaxes is likely lower than the flake scars generated during manufacture, as a portion of the piece is lost in the process. For example, a handaxe with 20 flake scars but of a smaller size is not definitely less retouched than a handaxe of larger size with 30 flake scars. Considering the number of scars in conjunction with the size of handaxes would make this attribute size-independent and improve its value. Coincidentally, in the analyses of core reduction intensity of some East African Oldowan sites, Braun et al. [36–38] also suggested that flake scar number divided by mass of the piece is a more appropriate measure of reduction intensity.

For exploring the use-life and implied human behaviors of handaxes (e.g., raw material transport), Shipton [39, 40] proposed a flake scar density index in his analysis of Indian and East African material. Specifically, the scar number on a handaxe is divided by the product of the handaxe length and width as an indication of the surface area. The principle here is that a handaxe will start off with a low flake scar density, and as the reduction progresses, the value of flake scar density will steadily increase [39–41]. This is a size-independent method which addresses the limitation of the scar number approach discussed above. Due to the imprecision in measuring surface area, Shipton et al. [42] then applied a 3D technique to capture the area more accurately, producing a 3D surface area. Simultaneously, Clarkson [43] used a similar method to measure the reduction intensity of different types of cores (with bifaces included as one core type), and he introduced the Scar Density Index (SDI, or the ratio of flake scar number to 3D surface area). Moreover, Clarkson [43], Clarkson et al. [44] and Shipton and Clarkson [41] have used both experimental and archaeological materials to reinforce the reliability of this index.

The purpose of this paper is to present a 3D quantitative analysis of reduction intensity of a handaxe assemblage from the Danjiangkou Reservoir Region (DRR), central China [45–47]. Here, reduction of the DRR handaxes includes both shaping and the probable re-sharpening process, as these two aspects cannot be objectively distinguished, especially in cases where the reduction intensity is generally low, as in DRR. In addition to the 3D Scar Density Index (SDI), a Flaked Area Index (FAI) which can quantify the reduction extent in different parts (i.e. tip, medial and base) of a handaxe, will also be used.

## Materials and Methods

### Materials

**Fig 1:**
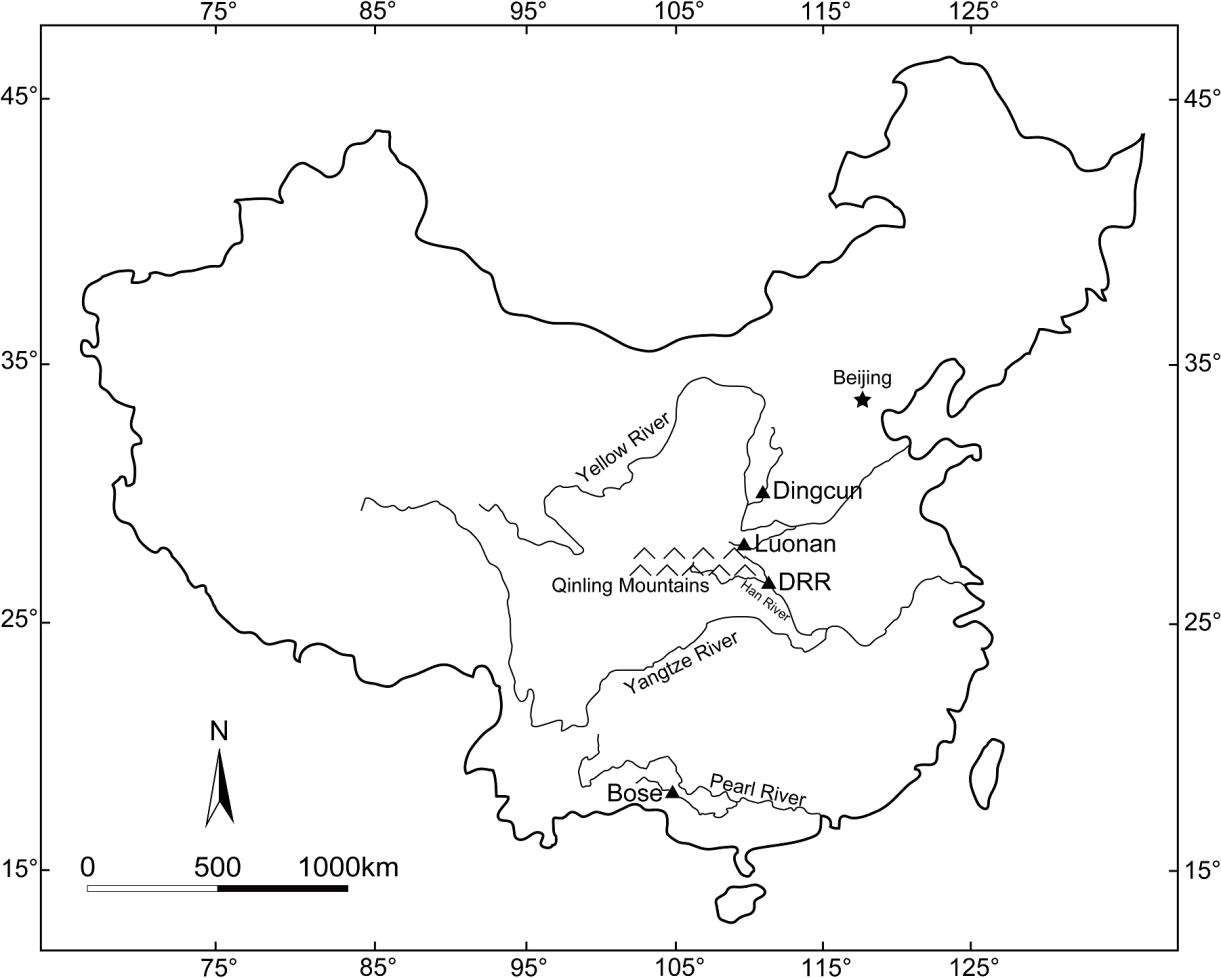
The Danjiangkou Reservoir Region (DRR) and the other well-known handaxe regions in China.

The handaxes analysed in this paper are from both surface collections and excavations on the third terrace (T3) of the Danjiangkou Reservoir Region (DRR), central China (Fig 1). Systematic investigations and excavations over the last two decades in this area have revealed it as another important handaxe-bearing region in China, along with the well-known regions of Dingcun, Bose and Luonan [45–59]. To ensure accurate measurements of surface area and volume for handaxes, only complete specimens are employed in our study, namely 92 handaxes in total (see S1 Table for raw data of each specimen). Of these, 76 are surface collected and 16 are excavated from the third terrace of the Han River, the longest tributary of the Yangtze River and the main feeder of the Danjiangkou Reservoir. The surface-collected material has been statistically confirmed to be consistent with excavated specimens in both morphology and technology [56]. In terms of the type of shaping of the DRR T3 handaxes, with the exception of one indeterminate specimen, 37 (40.2%) were bifacially shaped, 38 (41.3%) were partly bifacially shaped and only 16 (17.4%) were shaped unifacially (See S1 Table). The ESR, OSL and palaeomagnetic dating of the third terrace at the Shuangshu and Maling 2A sites indicate that these two handaxe-bearing sites formed in the Middle Pleistocene [51, 56].

In addition, 25 surface collected handaxes from the second terrace (T2) of the Han River were also used in a comparative study of differences in reduction intensity through time in the DRR (see S1 Table). The T2 Dishuiyan site is dated to ca 100–50 ka by the OSL and TT-OSL methods [60]. Here more than 20 handaxes comparable to the 25 surface-collected specimens used in this study were excavated.

### Ethics statement

The surface-collected handaxes (N = 101) were retrieved during field investigations carried out by one of the co-authors, Chaorong Li, and permission to study these materials was issued by the Institute of Vertebrate Paleontology and Paleoanthropology (Beijing), Chinese Academy of Sciences, in which these specimens are stored. The excavated handaxes (N = 16) are stored in the Danjiangkou Museum in Danjiangkou City, Hubei Province, and study of these materials was permitted by this museum. We ensure that the fieldwork did not involve endangered or protected species.

### 3D scanning and measuring

For capturing the 3D image of each handaxe, we used two types of laser scanners. The NextEngine 3D Laser Scanner was used in the field, as it is light and portable. To scan the whole surface of a handaxe, we conducted two separate scans in vertical and horizontal views which were subsequently merged into one complete 3D image. In the laboratory, the Range 7 3D Laser Scanner was used, as it gives excellent resolution but is difficult to carry in the field. Handaxes were rotated manually to obtain a complete 3D image. The mean value of triangles representing the degree of resolution is two times higher with the Range 7 scanner than with the NextEngine scanner. Holes on images were filled using the Geomagic Studio software, regardless of the scanner used.

**Fig 2:**
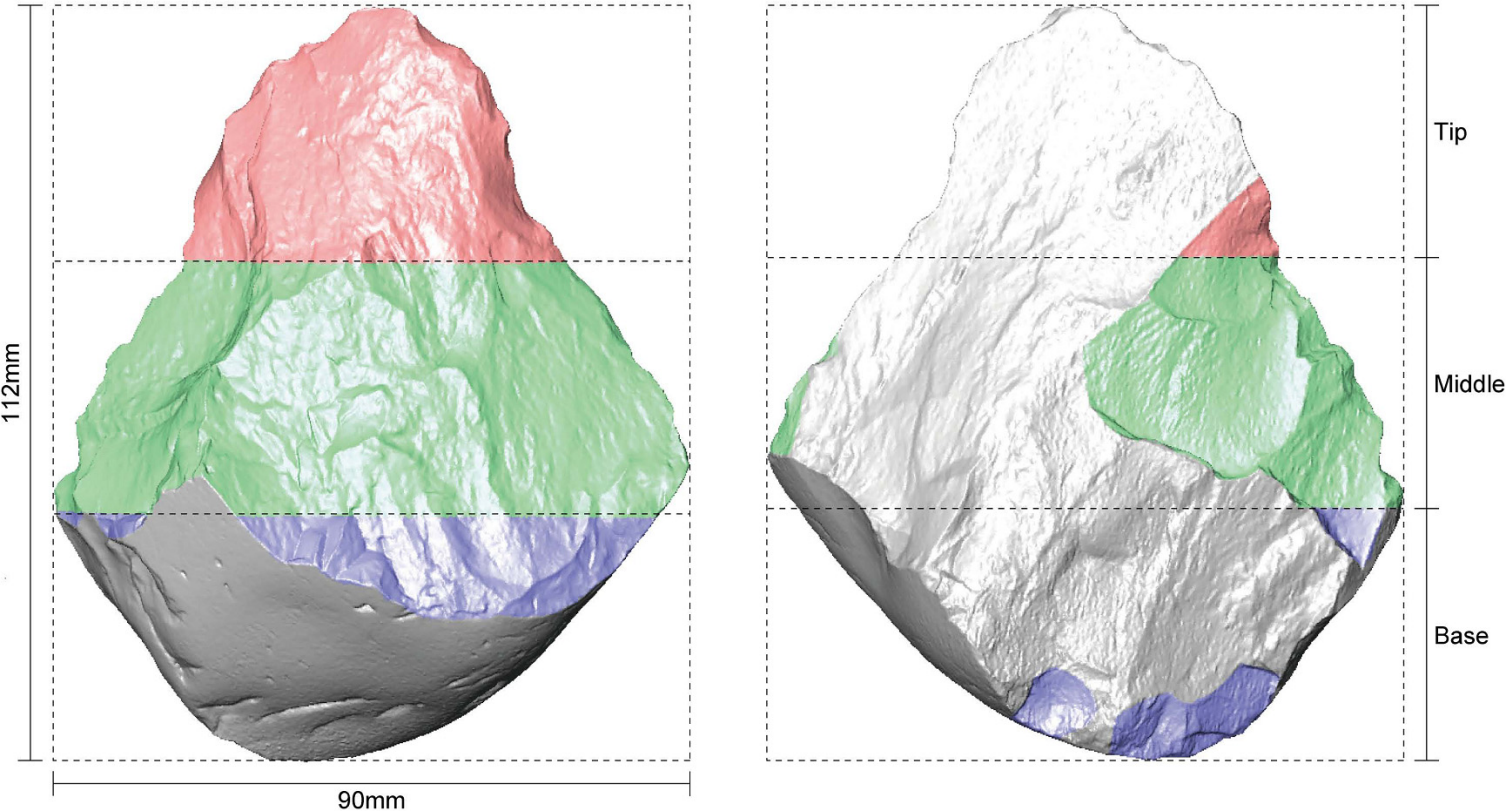
Segmentation of a 3D scanned handaxe (No. 1994, 93) into three proportional sectors.

After attaining the 3D images, we then imported them into the Avizo Fire 3D Imaging Software (version 8.0) to accurately calculate the surface area and volume of the handaxes. The segmentation function of this software was also applied to divide the handaxes into three portions with the piece aligned along the long axis, using the distal end as the guide. The scar coverage of each portion was then accurately extracted using this software. An example is given in Fig 2, which shows the proportional division of the surface into distal, medial and proximal parts based on the length, for which the area of flake scar coverage is then calculated in each sector.

### Indices of SDI and FAI

Flake scar density (equal to the Scar Density Index, SDI) has been used as an effective indicator of reduction intensity of Indian and East African handaxes [39–42]. In an experimental test of the reliability of SDI, the regression analysis of transformed log SDI and log % Mass Remaining of bifaces produced a very strong relationship (*R* ^*2*^ = 0.916) [43]. In addition, a recent study of the experimentally reduced handaxes also indicates a strong relationship (*R* ^*2*^ = 0.803) between increasing SDI and decreasing % original mass [41]. For this reason, the 3D SDI was adopted in our analysis of reduction extent of the DRR handaxes. All visible flake scars on a handaxe were counted in this study, regardless of their interpretation as shaping vs. refining scars. Because of the fresh condition of the DRR handaxes, it is easy to count the scar numbers. And because most of these handaxes were made on primary flakes or cobble opening flakes [46], dorsal scars on flake handaxes were mainly formed by the subsequent flaking. Therefore, all dorsal scars were counted as flaked area. However, the flaked area does not include the ventral surface of handaxes made on flakes if no scars are present.

As a result of the use of 3D technology, a new index, Flaked Area Index (FAI, flaked area divided by the total surface area), is now possible. A reasonable assumption for this index is that the flake scars area on handaxes increases with reduction. The unretouched blank for a handaxe would have a FAI value of 0, while a completely retouched handaxe would reach a FAI value of 1. Through the accurate measuring of the flaked area in different sectors of the handaxes, we can estimate not only the overall reduction intensity, but also the reduction intensity of the different parts, which is a benefit of FAI. We need to bear in mind that the flaked area does not necessarily relate to the number of flake scars. This is particularly applicable to hard hammer percussed handaxes, in which a small number of large scars can produce a large area of scar coverage, and conversely, a large number of small scars can produce a small area of scar coverage. Despite this, the FAI index can reflect the general pattern of the reduction extent of handaxes.

In addition, it should be noted that both the SDI and FAI indices will reveal the relative extent of reduction, but not the actual mass lost during the reduction. In order to investigate quantitatively how much mass has been lost in the reduction process, it is necessary to conduct knapping experiments in future research.

## Results

### The application of SDI to reduction intensity

**Fig 3:**
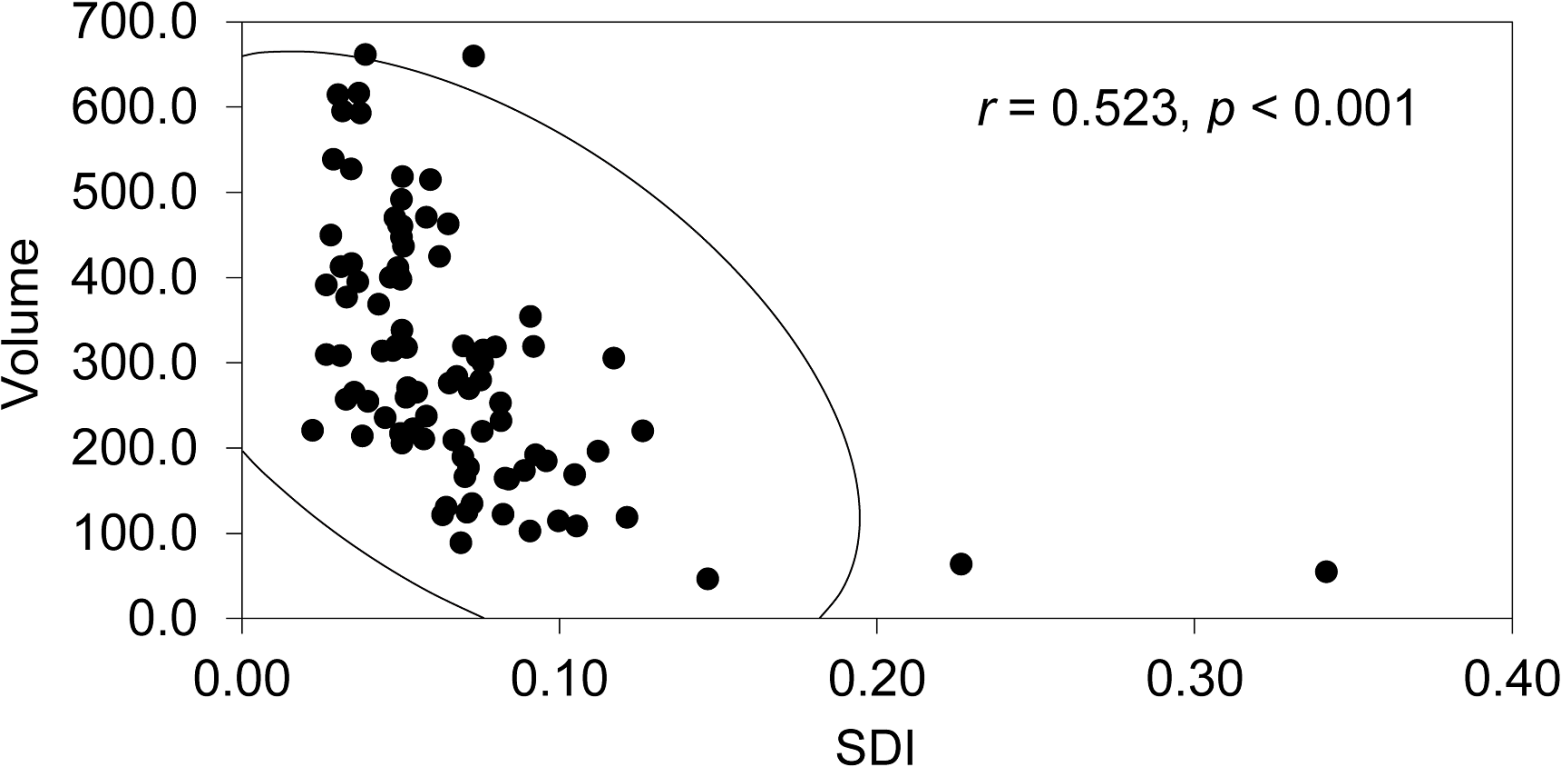
Correlation analysis between volume and SDI.

**Fig 4:**
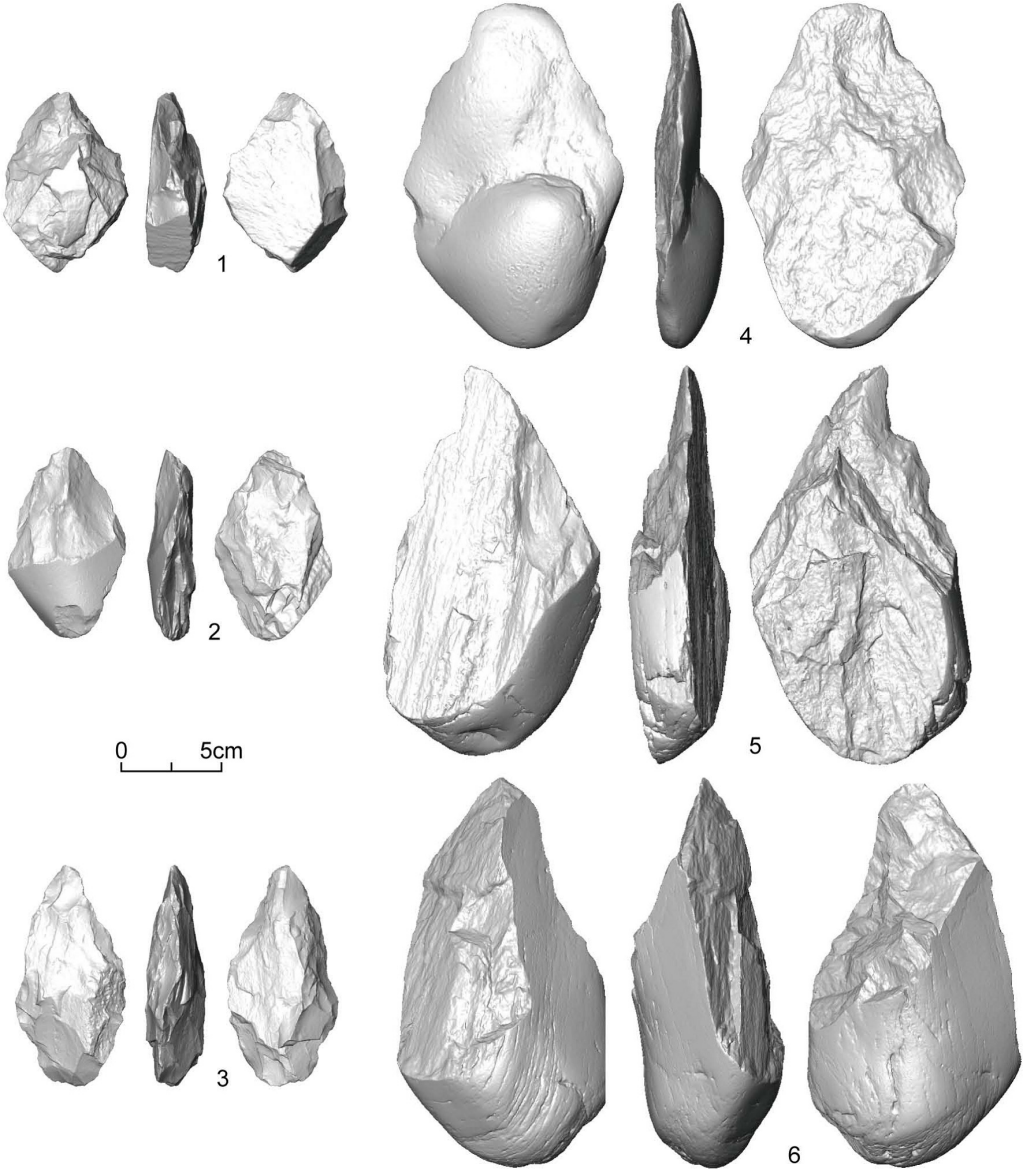
3D scans of the DRR handaxes from T3.

Correlation analysis between volume and SDI shows that the relationship between volume and SDI is significant (*r* = 0.523, *p* < 0.001), although there is considerable variation (Fig 3). In addition, to test the effect of outliers, we exclude specimens (N = 9) whose SDI values are larger than 0.1. Results show that the correlations between volume and SDI with and without outliers are very close to each other (*r* = 0.523 vs. *r* = 0.520). Therefore, the DRR handaxes can be confirmed to be made from similar size cobbles, and we suggest that the size-independent SDI used in this study is appropriate for measuring the reduction intensity of handaxes. Fig 4 shows three handaxes (left side of the figure) that are low in volume and weight but high in SDI, and three handaxes (right side of the figure) that are high in volume and weight but low in SDI. Detailed information for each specimen is provided in Table 1.

**Table 1:** Data for the DRR handaxes presented in Fig 4. See S1 Table for raw data.

Number	Provenance	Volume (cm^3^)	Weight (g)	Scars Number	Surface Area (cm^2^)	SDI	Total flaked area (cm^2^)	FAI
13	2004 onwards	63.8	168	24	105.9	0.227	70.7	0.668
150 *in situ*	1994	46.1	120	14	95.4	0.147	79.2	0.830
11	2004 onwards	54.9	144	37	108.3	0.342	108.3	1.000
SS-T3	Excavation	220.7	566	7	312.3	0.022	143.6	0.460
87	1994	391.1	1018	11	411.7	0.027	321.3	0.780
75	1994	538.3	1404	12	414.1	0.029	172.2	0.416

**Fig 5:**
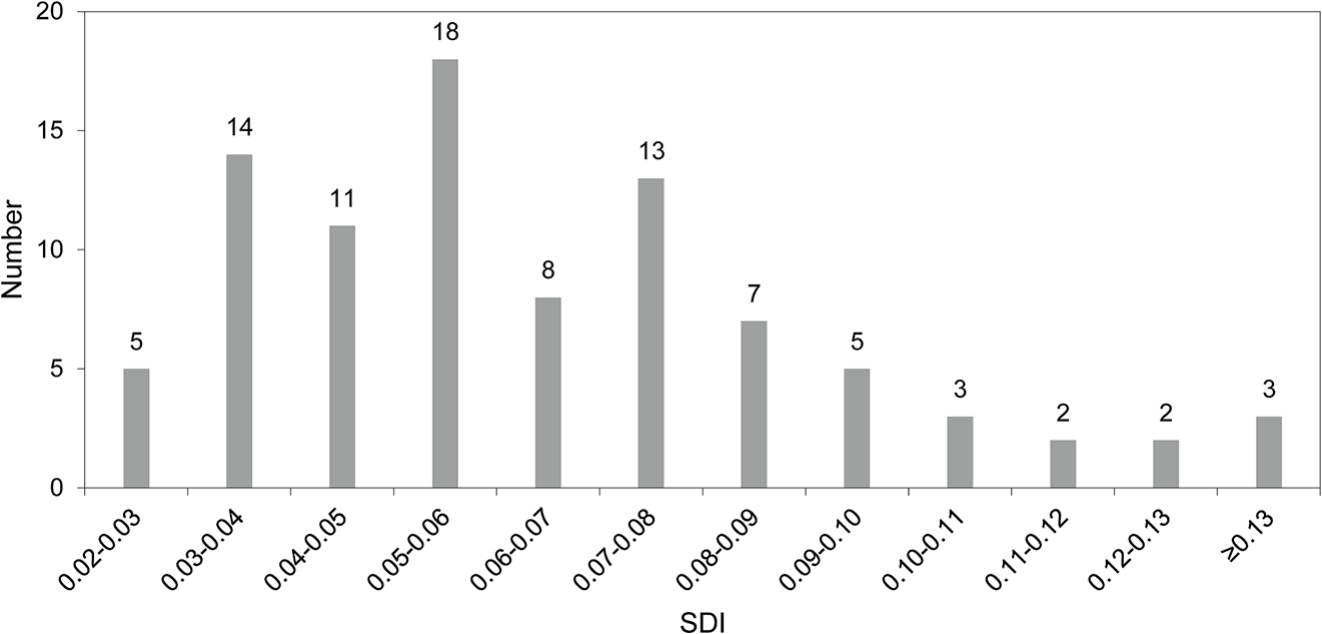
The number of handaxes within the different ranges of the SDI values.

**Fig 6:**
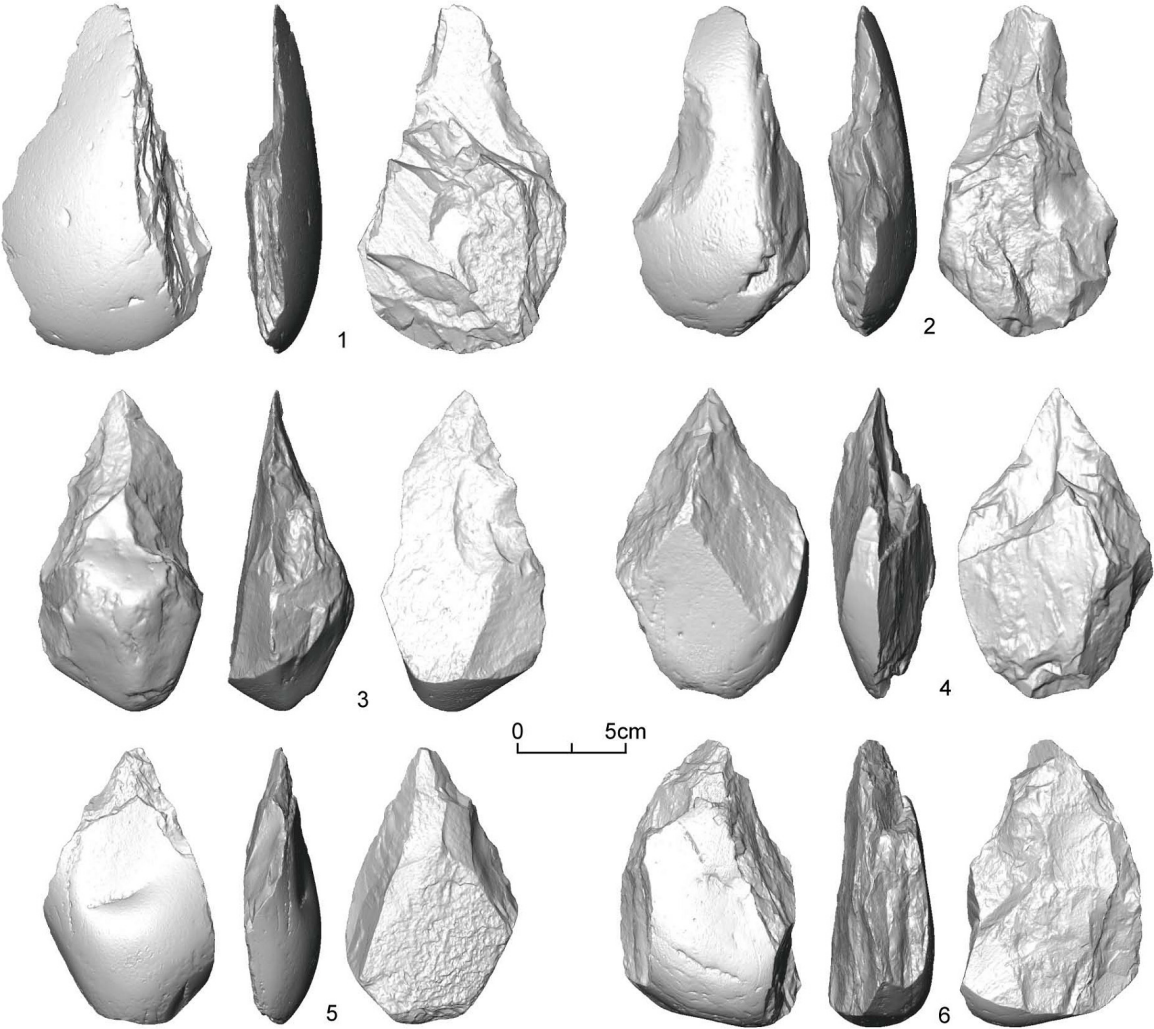
3D scans of the DRR handaxes from T3 with relatively high SDI values (0.08–0.09).

Although the indices of SDI used here cannot show how much mass is lost during reduction, they do provide us with information about the relative intensity of handaxe reduction. Adapting statistical models used in demography, Shott and colleagues suggest that different distributional patterns of reduction could correspond to different cumulative-survivorship curves [11, 61–63]. In other words, the fit between the length of handaxe use and SDI has the ability to reveal the underlying use-lives of tools and the related human behaviours. In Fig 5, we can see that most of the DRR handaxes possess lower SDI values and locate on the left side of the histogram, with 83.5% (N = 76) of them lower than 0.09 on the SDI value. Only a small number of handaxes has relatively higher SDI values and locate on the right side of this diagram. Therefore, it is reasonable to infer from Fig 5 that most of the DRR handaxes were less extensively reduced and generally had short use-life before discard. This situation may relate to the use of locally available raw materials close to the site and brief occupation periods. For visualizing the reduction intensity of the DRR handaxes, six handaxes with the values of SDI from 0.08 to 0.09 are presented in Fig 6 (see Table 2 for information on individual specimens).

**Table 2:** Data for the DRR handaxes presented in Fig 6. See S1 Table for raw data.

Number	Provenance	Scars number	Surface area (cm^2^)	SDI	Total flaked area (cm^2^)	FAI
2	2004 onwards	20	240.9	0.083	153.2	0.636
SS-546	Excavation	18	213.8	0.084	84.9	0.397
GCC-46+47	Excavation	21	257.8	0.081	121.2	0.470
70	2004 onwards	19	213.2	0.089	142.8	0.670
19	2004 onwards	14	170.1	0.082	55.9	0.329
23	2004 onwards	20	244.5	0.082	172.3	0.705

### The application of FAI to reduction intensity

**Fig 7:**
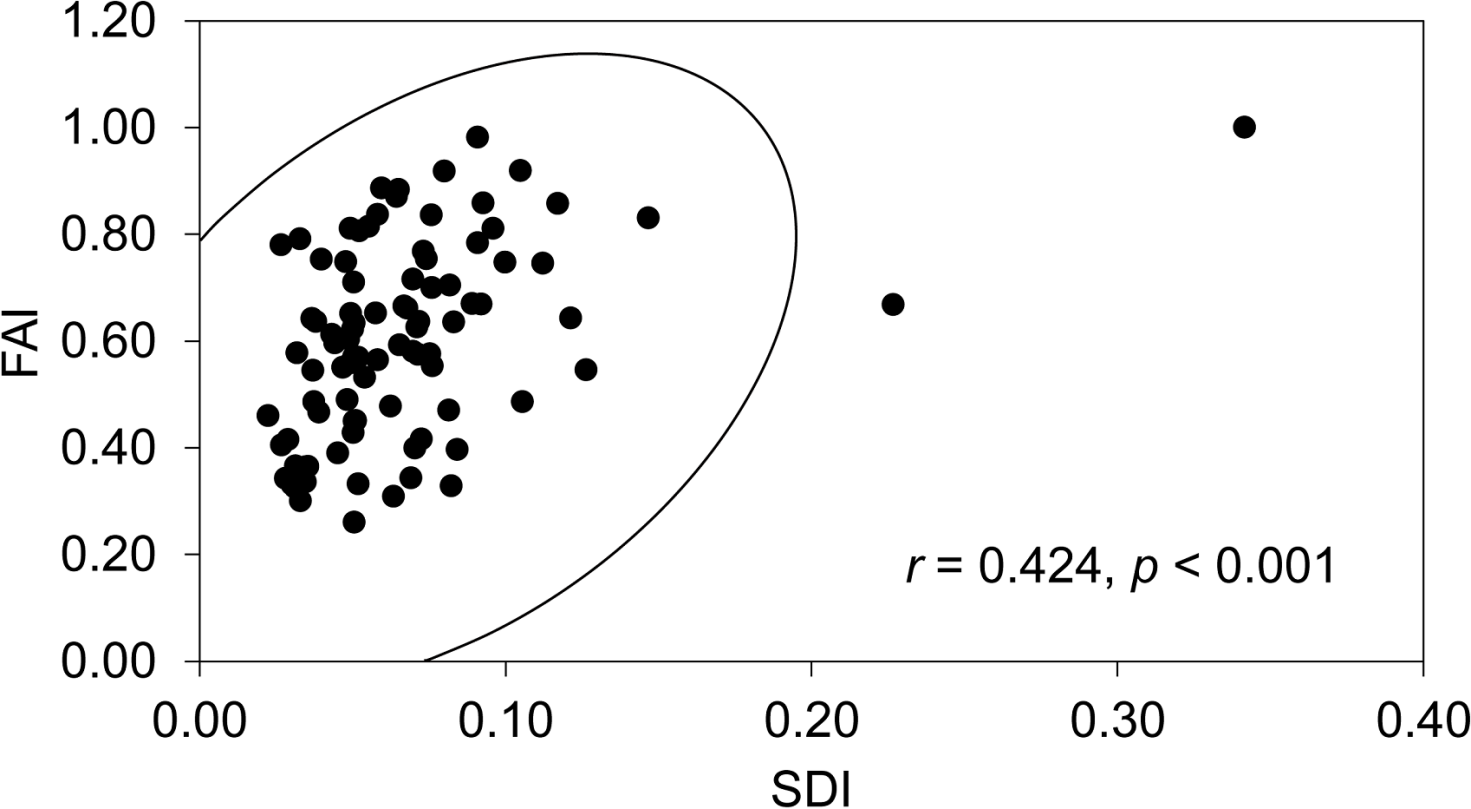
Correlation analysis between SDI and FAI.

**Fig 8:**
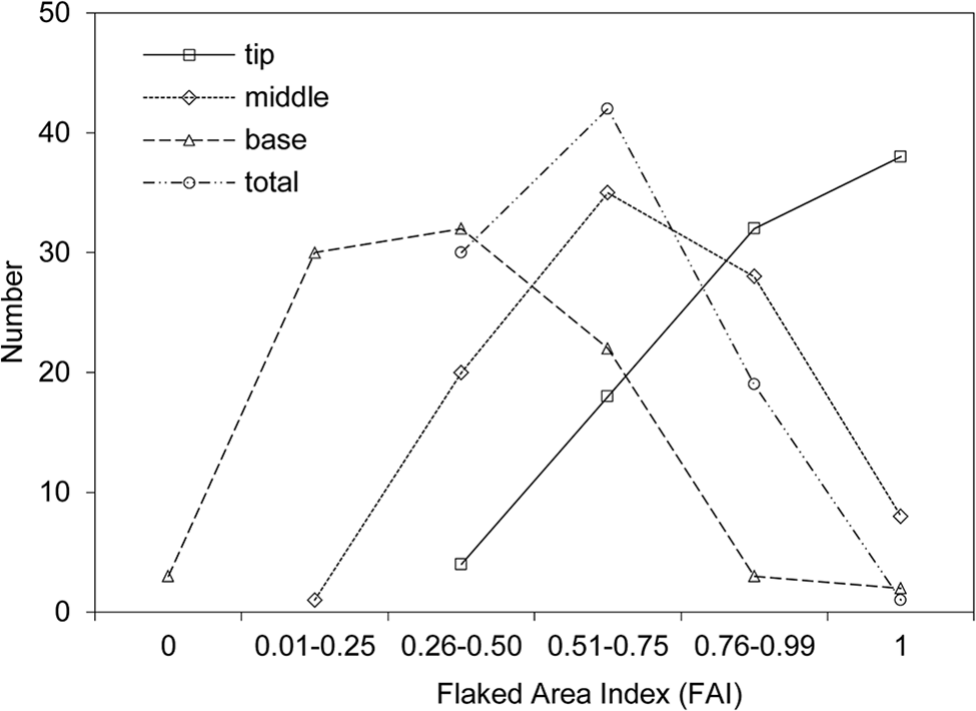
The distribution of the FAI values in relation to handaxe sectors.

Correlation analysis between SDI and FAI shows that these two indices are significantly related (*r* = 0.424, *p* < 0.001; see Fig 7), although there is considerable variation. This indicates the validity of FAI in estimating the reduction intensity of DRR handaxes. Based on the 3D segmentation of handaxes into three proportional parts, namely, tip, middle and base, we can examine the pattern of reduction intensity in each sector. Fig 8 and Table 3 show that the values of FAI for tips are high, with 34.8% of them scoring in the range 0.75–0.99 and 41.3% of them fully covered by flake scars. The mean value of FAI for tips is 0.87. For the middle sections, 59.7% are concentrated in the range 0.26–0.75 and 30.4% in the range 0.76–0.99, and the mean value for middle sections is 0.69. The FAI values for the bases are generally low, with 32.6% scoring in the range 0.01–0.25, 34.8% in the range 0.26–0.50 and 23.9% in the range 0.51–0.75, and a mean value of 0.37. Therefore, we can conclude that most of the shaping was invested in the tips of the DRR handaxes, while the bases usually have the least reduction, with an intermediate degree of reduction in the middle sectors. The reason for this pattern is likely related to the functional differences for each part: the tip is the most utilised part, while the middle may be related either to use or to shaping of the tip; and the smooth cobble surface is often left on the base for holding comfort. This conclusion is supported by using the sector method which records shaping status and calculates the proportion of shaping or cortex covered in each sector in the whole handaxe assemblage [45, 46]. Analysis of 36 handaxes found from 2004 onwards shows that shaping was mainly concentrated in the distal ends of DRR handaxes (86.8%), while the proximal ends were mainly occupied by cortex (50.0%), with a smaller percentage of shaping (38.9%; see Li et al. [46]).

**Table 3:** DRR handaxe analysis by sectors in relation to the FAI values.

FAI	Tip		Middle		Base		Total	
	*N*	*%*	*N*	*%*	*N*	*%*	*N*	*%*
0	0	0	0	0	3	3.3	0	0
0.01–0.25	0	0	1	1.1	30	32.6	0	0
0.26–0.50	4	4.3	20	21.7	32	34.8	30	32.6
0.51–0.75	18	19.6	35	38.0	22	23.9	42	45.7
0.76–0.99	32	34.8	28	30.4	3	3.3	19	20.7
1	38	41.3	8	8.7	2	2.2	1	1.1

The overall extent of reduction for the whole body of handaxes ranges from 0.26–0.99, with over 45.7% in the range of 0.51–0.75 (Table 3), and the mean value of FAI is 0.60. If a cut-off point of 0.75 is used to represent the boundary between extensive (> 0.75) and moderate reduction (≤ 0.75), we can see that more than three quarters (78.3%) of handaxes in DRR were only moderately reduced. This result is consistent with our analyses of the SDI, where results show that the DRR handaxes generally show a low extent of reduction.

### Comparing reduction intensity for different types of raw materials, blanks and shaping

**Fig 9:**
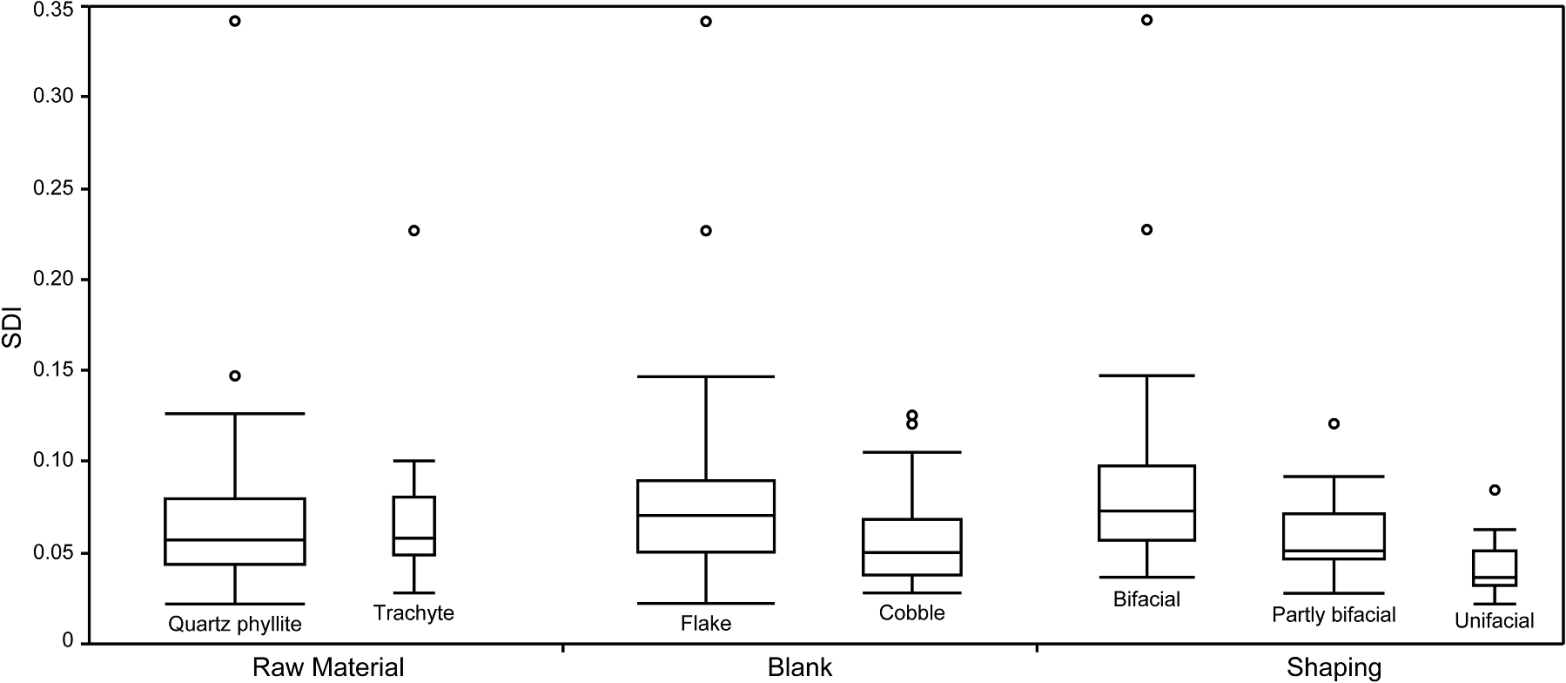
Box plots of SDI values for the different types of raw materials, blanks and shaping.

**Fig 10:**
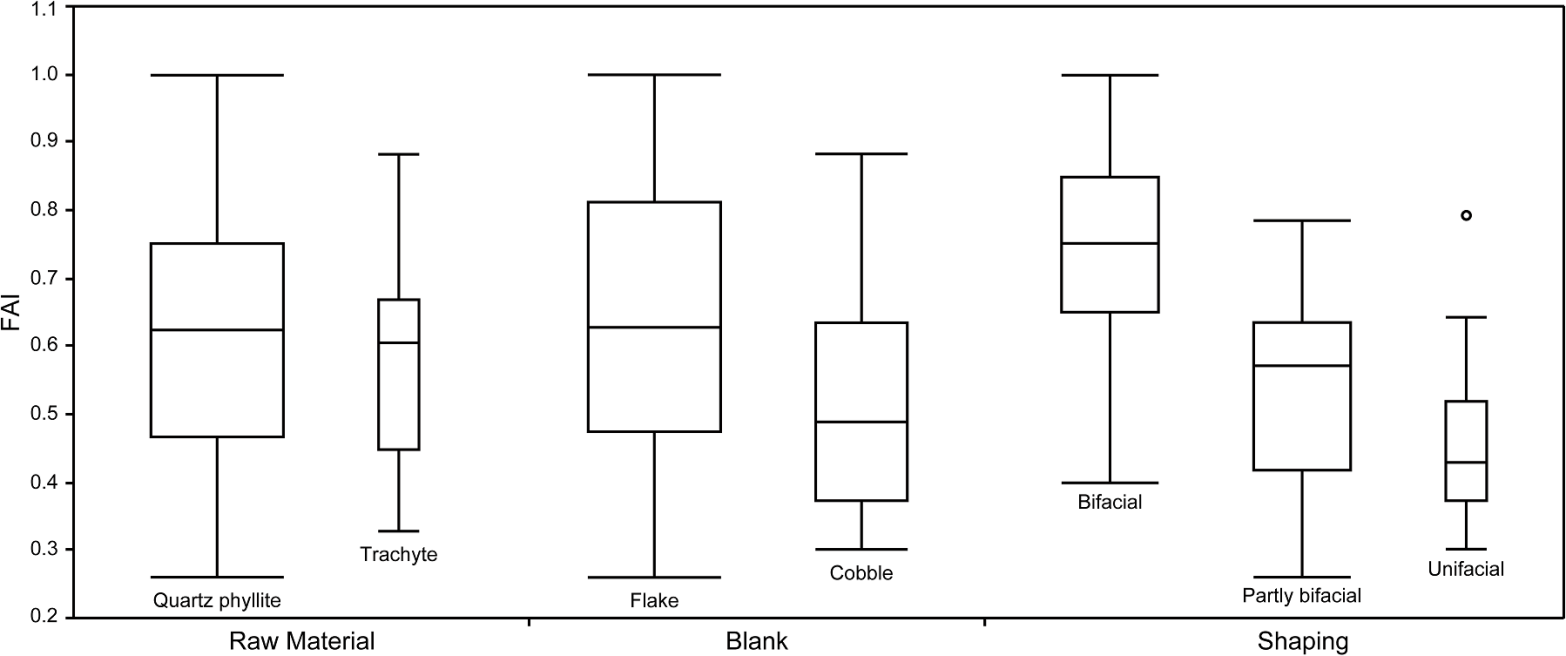
Box plots of FAI values for the different types of raw materials, blanks and shaping.

Quartz phyllite (N = 67) and trachyte (N = 15) were the most frequently used raw materials for DRR handaxes, although the number of trachyte pieces is much lower than the number of quartz phyllite in the current study sample (Table 4). Both raw materials are abundant and locally available in the nearby gravel layers of the Han River [50, 55, 56]. The comparisons of SDI and FAI by raw material show substantial overlap (left side of Figs 9 and 10). This observation is supported by the *t*-test (*t* = -0.305, *p* = 0.761 for SDI; *t* = 0.478, *p* = 0.634 for FAI), which shows there is no statistically significant difference in the levels of reduction between the two raw materials. We can, therefore, further infer that a consistent reduction strategy was employed despite raw material differences.

**Table 4:** Raw materials and blank types of the DRR handaxes from T3. Flake blanks include bipolar flakes.

	Quartz phyllite	Trachyte^a^	Other igneous rocks	Quartz	Indeterminate	Total
**Flake**	**32**	**8**	**4**		**1**	**45**
**Cobble**	**23**	**6**	**2**	**1**	**1**	**33**
**Split cobble**	**11**	**1**				**12**
**Indeterminate**	**1**	**1**				**2**
**Total**	**67**	**16**	**6**	**1**	**2**	**92**

^a^ Because of the highly weathered surface of one trachyte handaxe made on a cobble, its scar number is indeterminable. Thus, 15 trachyte handaxes were used for comparison. See S1 Table for raw data.

In contrast, there is a clear trend of lower SDI and FAI with cobble blanks (N = 32) compared with flake blanks (N = 45). This is visible on the middle part of Figs 9 and 10. The *t*-test also shows significant differences in the levels of reduction between the two blank groups (*t* = 2.438, *p* < 0.05 for SDI; *t* = 2.708, *p* < 0.01 for FAI). During the experimental test of the effects of blank type on handaxe reduction, Shipton and Clarkson [41] also noticed that cobble blanks tend to have lower SDI compared to flake blanks for a given percentage of mass lost. This difference in DRR T3 handaxes probably relates to the large flat ventral surface provided by flake blanks and their relative thinness compared to cobbles, both of which facilitate reduction.

In terms of the effect of types of shaping on the reduction intensity, the Kruskall-Wallis test shows that there are considerable differences for both SDI (ChiSquare = 25.6, *p* < 0.0001; see Fig 9) and FAI (ChiSquare = 39.0, *p* < 0.0001; see Fig 10) among the three shaping groups, namely, bifacial, partly bifacial and unifacial. Bifacially shaped handaxes have the greatest mean values of SDI (0.087) and FAI (0.743), while the unifacially shaped handaxes have the least mean values of SDI (0.042) and FAI (0.458), with partly bifacially shaped handaxes being intermediate (mean SDI = 0.058; mean FAI = 0.532). Therefore, it is clear that different types of shaping have a strong influence on the degree of reduction for the DRR T3 handaxes.

### Comparing the reduction intensity of handaxes from T2 and T3 of the DRR

The 3D quantitative method provides an objective way to compare reduction intensity through time. Here, the 25 handaxes from the second terrace of the DRR are compared with the 92 handaxes from the third terrace analysed in the foregoing sections. The preliminary age of handaxes from the second terrace is the early Late Pleistocene (100–50 ka) [60], while the handaxes on the third terrace are dated to the Middle Pleistocene [51, 56]. Therefore, handaxes in the DRR provide an opportunity to examine the regional change in reduction intensity from the Middle to the early Late Pleistocene. Because of the relatively small sample size from T2, we do not conduct a statistical analysis according to different types of raw materials, blanks and shaping, as was done for the sample from T3. Attributes used in this analysis include the number of scars, surface area, volume, total flaked area, and the indices of SDI and FAI. The *t*-test shows that there are no statistically significant differences (*p* > 0.05) between handaxes from T2 (the second terrace) and T3 (the third terrace) in any of these attributes (Table 5) and thus no technological change in reduction intensity through time in the DRR is presented.

**Table 5:** Comparison of mean values between handaxes from T2 and T3 in DRR.

	T2 (N = 25)	T3 (N = 91)^a^	*t*-test	*p*-value*
Scars number	16.76	18.12	-0.955	0.342
Surface area (cm^2^)	318.03	305.21	0.604	0.547
SDI	0.058	0.067	-0.969	0.335
Total flaked area (cm^2^)	196.85	181.69	0.882	0.380
FAI	0.620	0.605	0.384	0.702

^a^ Because of the highly weathered surface of one handaxe, its scar number is indeterminable. Thus, 91 handaxes were used for comparison. See S1 Table for raw data.

* *p* (two-tail) < 0.05 is a statistically significant difference.

## Summary and Conclusion

The extensive application of reduction intensity indices in the past three decades has remarkably improved the ability of lithic analysts to interpret human behaviour. With the aid of 3D scanning technology, in this paper we applied two quantitative reduction indices, the Scar Density Index (SDI) [41, 43, 44] and the Flaked Area Index (FAI), to the analysis of reduction intensity of the T3 DRR handaxes. The SDI in this study shows that most of the handaxes in DRR have a relatively low intensity of reduction, which also indicates a generally short use-life as argued by Shott and Sillitoe’s reduction distribution model [11, 61–63]. In addition, the short use-lives of these handaxes may suggest that open-air sites along the river terrace were not occupied by hominids for a long time. The analysis of FAI also shows that the overall reduction intensity of the DRR handaxes represents a least-effort reduction strategy, with 78.3% of handaxes only moderately reduced (FAI ≤ 0.75). The detailed FAI analysis of the different parts of the DRR handaxes shows that tips generally show the most reduction, while the bases show the least, a pattern which is relatively common in some Chinese handaxe assemblages. It is not surprising that the handaxe tip would receive most attention in shaping. The middle section could be functional if the edge were used, but it relates also to shaping of the piece overall. The base of a handaxe was the holding unit, with only limited or no shaping present.

The two raw materials used in DRR, both locally available, did not have an influence on the reduction intensity, suggesting that the behavioural interpretation of short-term use is correct. In addition and because of the abundance of raw materials, the DRR handaxe knappers seem to have employed an expedient exploitation strategy. However, the type of blanks and the shaping types did play a role in the reduction extent of the DRR handaxes. Those made on flake blanks generally show a higher level of reduction than those made on cobble blanks, presumably because flake blanks were thinner than cobbles, and they provided a large flat surface which made reduction easier. In terms of shaping, bifacially shaped handaxes show a greater degree of reduction than partly bifacial handaxes, with the unifacial handaxes showing the least reduction. The preliminary comparison of handaxes from T2 and T3 of the DRR suggests that there is no technological change in the reduction intensity from the Middle Pleistocene to the early Late Pleistocene in this region, although more specimens from terrace two need to be analysed.

The results presented here demonstrate that the application of quantitative technological indices is necessary and useful in estimating the reduction intensity of handaxes. According to this estimation, we can further investigate the behaviour of handaxe makers in the DRR, such as their adaptation to the local raw materials, their energy investment in making handaxes, and the use-life of handaxes. The potential of the indices used in this paper has been confirmed; however, as we have mentioned already, these indices can only indicate the relative extent of handaxe reduction and they still need to be further tested. In future research, experiments will be conducted to further evaluate the mass lost at different levels of the index values, and to support the validity of the current indices. Additionally, owing to the long lasting and widespread use of handaxe technology in the Pleistocene, the reduction intensity of handaxes at different developmental stages and in different regions will be further examined to address the technological evolution and adaptive behaviour of Acheulean hominids. Finally, this study has provided detailed information on the nature of handaxes in the DRR, which will serve as a comparative sample for a better overall understanding of these industries in China, in comparison with the western Acheulean.

## Supporting Information

S1 Table Raw data of the DRR handaxes from both terraces. (XISX).
(XLS)
